# Multiple invasions decimate the most imperiled freshwater invertebrates

**DOI:** 10.1007/s10530-025-03540-5

**Published:** 2025-02-13

**Authors:** Alexander Y. Karatayev, Lyubov E. Burlakova, Vadim A. Karatayev, John E. Cooper, Lars G. Rudstam

**Affiliations:** 1 Great Lakes Center, SUNY Buffalo State University, 1300 Elmwood Ave., Buffalo, NY 14222 USA; 2 Aquatic Ecology, Swiss Federal Institute of Aquatic Science and Technology (Eawag), Überlandstrasse 133, 8600 Dübendorf, Switzerland; 3 Department of Biology, University of Maryland College Park, Maryland, USA; 4 Cooper Environmental Research, 1444 County Route 23, Constantia, NY 13044 USA; 5 Cornell Biological Field Station and Department of Natural Resources and the Environment, Cornell University, Ithaca, NY 14850 USA

**Keywords:** Invasive species, *Dreissena*, Gastropoda, Molluscs, Round goby, Ecological impact

## Abstract

Invaders can have devastating impacts on freshwater ecosystems, but these impacts can subside over time as ecosystems “adapt” to the invasion of new species. We analyzed changes in species composition and density of molluscs in Oneida Lake (New York, USA), one of the best studied North American lakes based on detailed surveys conducted in 1915–17, 1967–68, 1992–95, 2012, and 2022–23, and on annual benthic surveys from 2009 through 2023. Eutrophication and habitat alteration after 1920 resulted in a 25% decline in species richness and a 95% decline in the density of native gastropods by 1967, while species richness of unionids did not change. The arrival of zebra mussels in 1991 and quagga mussels in 2005 was associated with an increase in species richness and density of native gastropods and an extirpation of unionids by 1995. However, an invasion by the round goby in 2013 led to a significant decline across all gastropod families, disproportionately impacting soft-shelled and shallow-dwelling species, while other species, including invasive dreissenids, partially recovered 3–7 years after the goby invasion. This mollusc recovery was depth-related and was limited to deeper areas. Altogether, molluscan communities were sensitive to ecosystem change and invasives species, with some invaders offsetting the impacts of eutrophication and habitat alterations. While individual stressors have taxon-specific and sometimes positive impacts, eutrophication and species invasions have collectively decimated the native mollusc community over the past century.

**Supplementary Information:**

The online version contains supplementary material available at 10.1007/s10530-025-03540-5.

## Introduction

Freshwater ecosystems occupy < 1% of the Earth’s surface but are considered hotspots for both human activity and biodiversity (Kummu et al. 2011; Strayer and Dudgeon 2010; Vörösmarty et al. 2010; Dudgeon 2019). Increasing anthropogenic pressure, including habitat destruction, habitat fragmentation, pollution, introduction of invasive species and climate change make freshwaters the most endangered ecosystems on Earth (Strayer and Dudgeon 2010; Dudgeon 2019). Freshwater molluscs are particularly affected by human impacts making them the most endangered animals world-wide (Lydeard et al. 2004; Bogan 2008; Johnson et al. 2013; Lopes-Lima et al. 2017, 2018). In North America over 75% of unionids and 74% of gastropods are imperiled (vulnerable, threatened, endangered) or extinct (Bogan 1993; Johnson et al. 2013). Although freshwater ecosystems suffer from multiple types of human activities, habitat destruction and introduction of invasive species are often the most common factors causing deterioration of native communities and extirpation of species (Wilcove et al. 1998; Dudgeon et al. 2006; Bogan 2008; Lopes-Lima et al. 2018).

The highest impact of invasive species usually occurs in the initial phase of the invasion when invader population are growing and high (Karatayev and Burlakova 1995; Strayer et al. 2017; Karatayev et al. 2015, 2023). Later in the invasion, the impacts of species introductions can subside due to changes in community structure, density-dependent changes in the abundance of the invader, or evolutionary and behavioral adaptations (learning, eco-evo dynamics) by resident species to the new invader (Simberloff and Gibbons 2004; Karatayev et al. 2015, 2023; Strayer et al. 2017). However, the time course of ecosystem impact and recovery may be disrupted if multiple species are introduced in series (e.g. invasion meltdown, Simberloff and Von Holle 1999; Karatayev et al. 2023).

The zebra mussel (*Dreissena polymorpha* (Pallas 1771)) and quagga mussel (*D. rostriformis bugensis*, Andrusov 1897) represent a novel ecological type in many freshwater systems and are causing dramatic impacts on the invaded ecosystems. As attached bivalves that are also highly efficient suspension feeders, dreissenids are powerful ecosystem engineers that modify bottom substrates creating complex reef-like habitats for benthic invertebrates, increase light penetration, and redistribute energy flows from pelagic to a benthic environment (Karatayev et al. 2002, 2015, 2023; Mayer et al. 2002, 2014; Gutierrez et al. 2003; Zhu et al. 2006; Sousa et al. 2009; Higgins and Vander Zanden 2010; Karatayev and Burlakova 2022a, b). As zebra mussels exhibit faster landscape-level spread, quagga mussels typically invade waterbodies with thriving zebra mussel populations (Karatayev et al. 2011, 2015; Strayer et al. 2019; Hetherington et al. 2019). This second dreissenid invasion amplifies the initial zebra mussel invasion impact (Barbiero et al. 2018; Karatayev et al. 2023).

The round goby (*Neogobius melanostomus* (Pallas 1814)) is another Ponto-Caspian invader actively spreading across Europe and North America since the 1990s (Wolter and Röhr 2010; Kornis et al. 2012; Schomaker and Wolter 2014) and invading waterbodies already colonized by both dreissenid species. As a benthic feeder, the round goby consumes bottom invertebrates and various molluscs including dreissenids, being adapted to feed on dreissenids through their co-evolutionary history (Kornis et al. 2012; Rudstam and Gandino 2020; Karatayev et al. 2024).

Oneida Lake is one of the best studied lakes in North America with continuous long-term fisheries and limnology data set since 1975 (Rudstam et al. 2016), including benthic invertebrates, dreissenid mussels, and round goby (Hetherington et al. 2019; Brooking et al. 2022). It is therefore an ideal system to evaluate ecological changes associated with invasive species. Through competition from dreissenids and predation by gobies, invasions may have strong impacts on native mollusc communities of invaded waterbodies. Mayer et al. (2002) found that the arrival of zebra mussels in 1991 led to increases in several benthic invertebrate groups which they attributed to ecosystem engineering by zebra mussels that increased the light levels on the bottom and associated benthic production (Zhu et al. 2006; Cecala et al. 2008), in addition to moving pelagic energy to the benthos and providing refuge habitats from fish predation—processes collectively referred to as benthification (Mills et al. 2003; Mayer et al. 2014). Karatayev et al. (2014) studied the changes in the Oneida mollusc fauna using a 100-year time perspective and found that dreissenid mussels reversed the decline in gastropods caused by eutrophication in the 1950s and 1960s. These ecosystem effects attributed to zebra mussels increased after the arrival of quagga mussels in 2005 (Karatayev et al. 2023). Round gobies were first reported in Oneida Lake in 2013 and were abundant by 2015 (Brooking et al. 2022). As in other lakes (Kornis et al. 2012), Oneida Lake gobies feed on benthic invertebrates, with dreissenids being the most abundant prey item in gobies larger than 70 mm (Poslednik et al. 2023). The arrival of the round goby may threaten the remaining mollusc community, especially species which have thinner shells or inhabit areas with high goby densities.

Here, we examine how 100 years of eutrophication and species invasions altered the species composition and spatial distribution of the mollusc community in Oneida Lake. We first present the lake-wide mollusc surveys conducted over the last 100 years and compare them with a similar lake-wide survey in 2022–2023, about a decade after the arrival of the round goby. We also use species-level information on the gastropods in intensive annual surveys conducted in the fall of each year since 1992 (Hetherington et al. 2019) to resolve the time trends in the Oneida mollusc fauna across depths and compare the effect of eutrophication and invasions on each mollusc group. We specifically test the following hypotheses: (1) goby invasion will decimate the mollusc community, but the effect will depend on species, with the soft-shelled species being affected the most, (2) predation effect will be size- and depth-dependent, and (3) with time molluscs will recover, but the degree of recovery will be species- and depth-dependent.

## Methods

### Field sampling

**Fig. 1:**
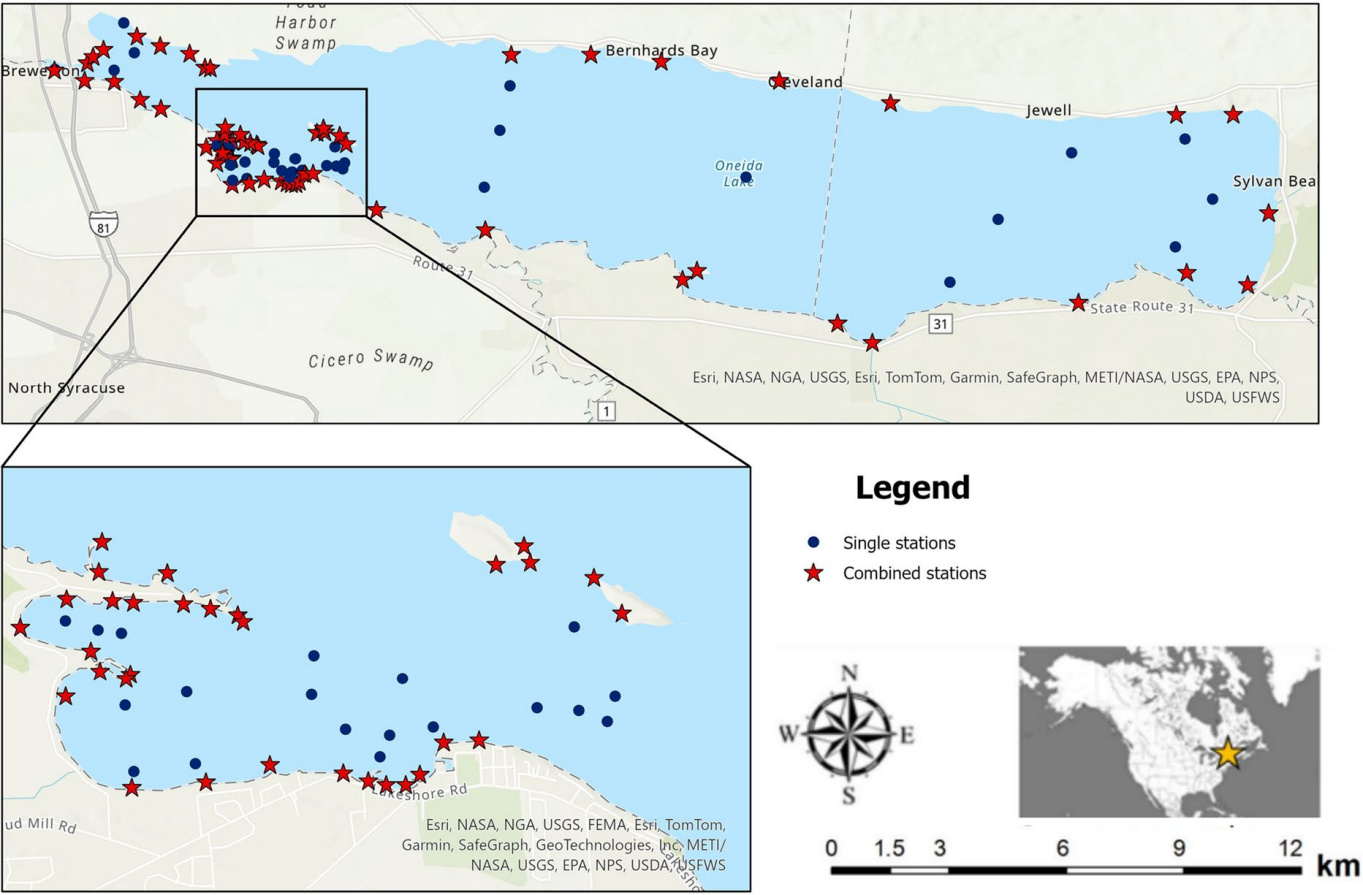
Map of locations in Oneida Lake sampled in 2022 and 2023. Each nearshore location (stars) included quantitative samples at 0.1–0.3 m, 0.5 m, and 1 m depths, and a qualitative sample. The inset provides sampling locations in the Lower South Bay from which samples were collected in 1917, 1967, 1992, 2012, and 2022, and used in the historical analysis of the mollusc community

Since the early twentieth century, the molluscan community of Oneida Lake was studied multiple times, including 1915–17 (Baker 1916, 1918), 1967–68 (Harman and Forney 1970), 1992–95 (Harman 1993, 2000), 2012 (Karatayev et al. 2014), and 2022–23 (this study) and annual benthic surveys from 2009 through 2023 (this study). Historically, the most detailed studies were conducted in the Lower South Bay (LSB) of the lake (Fig. 1). Therefore, across the paper we presented data separately for the LSB and lake-wide (the whole lake, including LSB).

#### 2012 and 2022–23 lake-wide surveys

We used identical sampling protocols in both the 2012 and the 2022–2023 surveys, including samples from stations in the littoral and profundal zones across Oneida Lake (Karatayev et al. 2014). The most recent lake-wide survey was conducted in August of 2022 (LSB) and July–August 2023 (rest of the lake). One to three replicate samples were collected from 86 stations in the LSB, and from 123 stations located in other parts of the lake (Online Resource 1). Our stations were at the same locations as in previous studies, including sites from a qualitative mollusc study in westernmost parts of the lake conducted by Baker in 1915 (Baker 1916), quantitative studies of LSB by Baker in 1917 (Baker 1918), and by Harman and Forney in 1967 (Harman and Forney 1970). For the 1967 survey, Harman and Forney (1970) reported the average density for each species across all samples and only presence-absence data for each species in samples collected in 1967 were available (provided by Harman).

Although qualitative samples in 1968 and 1990s (Harman 1993, 2000) were also collected around the lake, we were unable to determine the locations of these sites (Karatayev et al. 2014). Therefore, our 2012 and 2022–2023 stations in the central and eastern areas of the lake were located at random intervals along the shoreline (20 locations), and along 4 north–south transects in the profundal (> 7 m) zone where 123 quantitative samples were collected (Fig. 1).

For the 2012 and 2022–23 surveys, we collected samples at the water boundary (0.1–0.3 m), 0.5 m, and 1 m depths at each nearshore location to capture the diversity and distribution of molluscs in this physically heterogeneous area of the lake (Online Resource 1). In addition, molluscan species composition was examined qualitatively at most of the nearshore locations along a 30–50 m stretch of the littoral zone down to a depth of 1 m. On rocky substrates, samples were collected by scraping molluscs from rocks (surface area 0.01–0.12 m^−2^), while Ekman grabs (0.023 m^−2^) were used to sample soft and unconsolidated sediments. Samples of emergent macrophytes were collected by harvesting vegetation from a known area (usually about 0.02 m^−2^), including roots and sediments. At each profundal location 1–3 replicate samples were collected using an Ekman grab. All samples were washed through a 500 mm mesh, stored in sealable plastic bags, and transferred to the lab. Within 48 h after collection all molluscs were picked live from the samples. In 2012, right after picking, all dreissenids were identified to species, counted, and weighed; all other molluscs were preserved in 10% neutral buffered formalin. In 2022–2023, dreissenids were preserved together with other molluscs. Later in the lab, all molluscs were identified, and the density and biomass (total wet weight, with shells) for 2012 and 2022–2023 were determined for every species in each sample. Wet weight was determined after blotting off excess water. All molluscs collected were identified to species except for sphaeriids and *Physella*, which were identified to genus level.

#### 2009–2023 annual mussel and gastropod surveys

Dreissenid mussels have been sampled annually in the fall each year since 1992 by Ed Mills and colleagues (Mellina et al. 1995; Mayer et al. 2016; Hetherington et al. 2019). Briefly, Ekman grabs were used in soft and sandy substrates and divers scraped rocks found within a quadrat in rocky substrates. Between one and three replicates were collected at each site, filtered with a 253-µm mesh screen and then frozen for later identification in the laboratory. Since 2009, gastropods were also identified and enumerated. Between 21 and 60 stations were sampled each year from 2009 to 2023, mainly at sites between 2 and 11 m depths. Due to changes in the sampling design, depths shallower than 2 m were not sampled before 2015, depths deeper than 7 m were not sampled in 2009, and depths deeper than 11 m were not sampled until after 2013. All gastropods from the samples were removed and identified to the lowest practical taxonomic unit, mostly species, and counted. Small hydrobiids and amnicolids were lumped together as Hydrobiidae. The species identified are listed in Online Resource 2. For our analysis, replicate samples from a site were averaged and site was used as the elementary sampling unit. Note that gastropods were not measured or weighed and only density data is available for this group. Dreissenid mussel lengths and shell-on dry weights were measured. Details on the sampling procedure are in Hetherington et al. (2019) and in the metadata accompanying the web-based data repository (Rudstam and Almeida 2023). A map with the sampling locations is in Hetherington et al. (2019). The mussel and gastropod data up to 2019 were analyzed by Brooking et al. (2022). Here we use 2009 to 2023 data on specific gastropod species to test for depth and family-specific changes associated with the arrival of the round goby. These annual surveys do not include samples in LSB.

### Taxonomic resolution

**Fig. 2:**
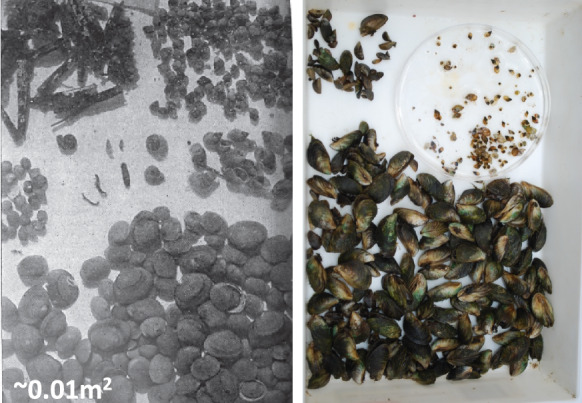
Photo of one of the Baker’s samples from Oneida Lake (from Baker 1918) (left panel) and of a current sample (right panel). Baker wrote: “Note the great abundance of the bivalve mollusc *Sphaerium* and the gastropod *Amnicola*”. The formerly abundant large sphaeriids are now replaced by dreissenids

With any long-term analysis of a community, it is important to consider changes in taxonomic resolution over time. In 1915–1917, famous taxonomist Frank Collins Baker conducted a thorough survey of Oneida Lake molluscs and published results of his study in two books with detailed description of sampling sites, species description and distribution, and provided actual photos of multiple samples (Baker 1916, 1918). His survey was one of the first quantitative studies of freshwater molluscs in North America. In taxonomic terms, Baker was a splitter (Harman and Forney 1970). During three years of his research, he recognized and named 92 species and subspecies of molluscs in Oneida Lake, including 30 species of Sphaeriidae (e.g. large-bodied *Sphaerium* spp. and *Musculium* spp.; Fig. 2), and 9 species of *Amnicola* (Online Resource 2). Many of these species later were synonymized into fewer species (e.g. all Baker’s *Amnicola* species were lumped into *Amnicola limosus* (Say 1817) and *Marstonia lustrica* (Pilsbry 1890)). Due to problematic taxonomy of Sphaeriidae beyond genus level, both 1967–1968 and 1993–1995 studies excluded this group from the analysis. To avoid taxonomic confusion, we lumped all species of Sphaeriidae into genera *Sphaerium, Musculium,* and *Pisidium*, and used their density for our 2012 and 2022–2023 study. We excluded *Margaritifera margaritifera* (Linnaeus 1758) from Baker species list as he later considered this specimen to be *Elliptio complanata* (Lightfoot 1786; Baker 1918). We converted data reported in all studies to common taxonomic resolution according to the Integrated Taxonomic Index System (http://www.itis.gov). Based on the origin, we distinguish native species, native transplant (introduced by humans from another region on the same continent) and exotic (from a different continent or major biogeographic region) species. The taxonomy for the 2012 and 2022–23 surveys was checked by AYK. The species identification in the annual surveys was done by JEC for all years included (2009–2023). The species identified by JEC and AYK were brought to the same level of taxonomic resolution (Online Resource 2).

### Data analysis

#### Pristine conditions, eutrophication and Dreissena invasion

To test whether community dominance changed among years, we analyzed k-dominance curves and conducted “Similarity profile” permutation tests (SIMPROF routine) on the overall gastropod density data from 1917, 1967, 2012, and 2022 in LSB. The k-dominance curves were determined by ranking species in decreasing order of overall abundance along the x-axis, with their relative contribution to the cumulative gastropod abundance on the y-axis. We used cluster analysis to distinguish assemblages among years, followed by SIMPROF to test for significant differences among clusters. The data was square-root transformed for analysis. To visualize the differences among assemblages, we used Non-metric Multi-Dimensional Scaling (NMDS), which calculates a set of metric coordinates for each sample that most closely approximates the nonmetric distance to every other sample based on Bray–Curtis similarity indices. These multivariate tests were performed in Primer software (version 7, Primer-E Ltd, Plymouth, UK). All test effects were considered significant if *P* < 0.05.

#### Goby invasion

We used the annual surveys to track the effects of goby invasion and depth on gastropod densities over time for each of the most abundant gastropod families using 2-dimensional splines in the R package ‘mgcv’ (Wood 2017). Our use of splines accounts for a potentially nonlinear effect of predictors (e.g. time or depth). We chose the complexity of each spline (i.e. basis dimension) based on AIC. As different families had very different mean densities, we rank-transformed the density of each gastropod family across sites and years in order to more easily compare trends across families.

To visualize trends, our core models tracked gastropod rank density by family as a function of depth and year. To compare the importance of year, depth, round goby density (expressed as a Round Goby index), and year since goby invasion, we also fit a set of 7 models and compared models via AIC. The Round Goby index was obtained from Brooking et al. (2022, updated in VanDeValk et al. 2024) and was calculated based on three standard sampling gears (bottom trawl, seine, and fyke-net, all years) and an annual video survey since 2018 (for details see Brooking et al. 2022). We judged model performance as significantly different when the AIC difference between models was > 2.

To compare the changes in density, biomass, and average weight (calculated as the total wet biomass divided by the density) at stations sampled in 2012 and 2022–2023, we used two-sided *t*-tests in Statistica (TIBCO Software Inc. (2020), Data Science Workbench, version 14. http://tibco.com). Depth-weighted lake-wide average density and biomass of molluscs were calculated from average densities and biomass at each depth zone (< 0.3 m, 0.3–3.0 m, 3.0–7.0 m, and > 7 m) weighed by the proportion of the total lake area represented by each zone (1.03%, 17.26%, 31.68%, and 50.03%, respectively) (Manly 2009). To compare the distribution of mollusc densities by depth zones between 2012 and 2022–2023, we used Fisher’s exact test with asymptotic p-value calculated based on chi-square distribution.

## Results

### Pristine conditions

**Fig. 3:**
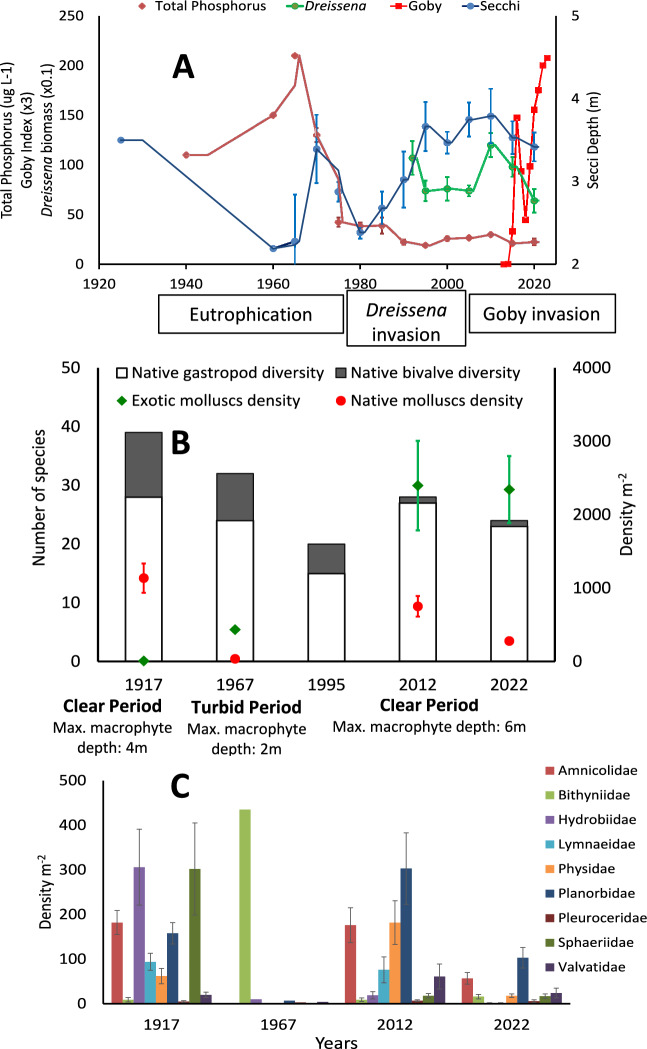
Long-term changes in **A**: Secchi depth, total phosphorus (μg/L), *Dreissena* wet biomass and round goby index in Oneida Lake. Values, except goby index, are calculated based on annual May–October averages for each five-year period to smooth trends. Annual data and methods are available in Rudstam and Almeida (2023) and VanDeValk et al. (2024). Data for years prior to 1960 are from Fitzgerald et al. (2016) based on grey literature reports. The phosphorus values from 1960s are soluble reactive phosphorus and therefore represents a minimum value for TP in the lake at that time. **B**: Lake-wide native gastropod and bivalve diversity and density (± standard error, where available) of all exotic and native molluscs in the Lower South Bay, Oneida Lake. **C**: Densities of major families in the Lower South Bay

**Fig. 4:**
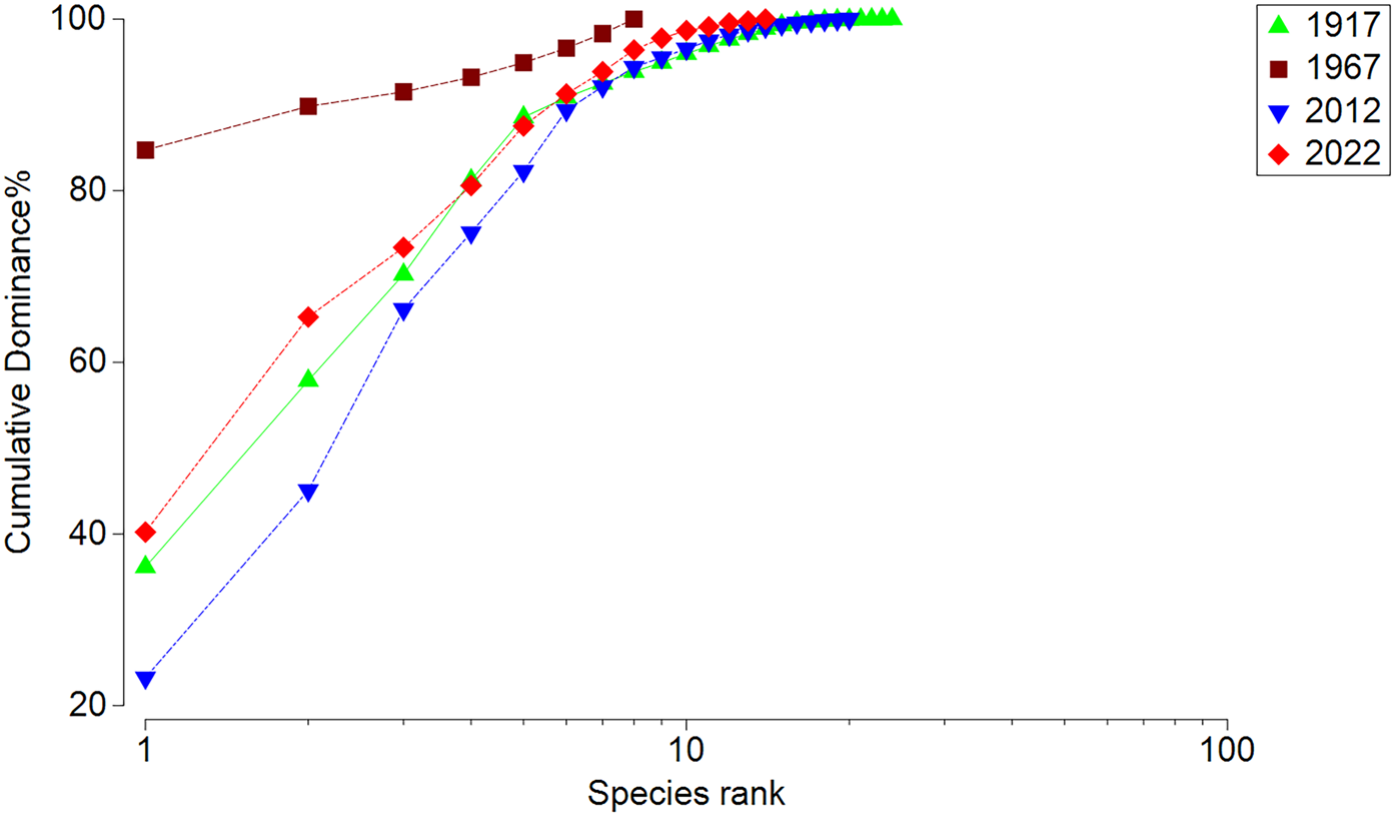
Dominance curves of gastropod assemblage in the Lower South Bay of Oneida Lake in 1917, 1967, 2012, and 2022 based on species densities across all samples in each study

**Fig. 5:**
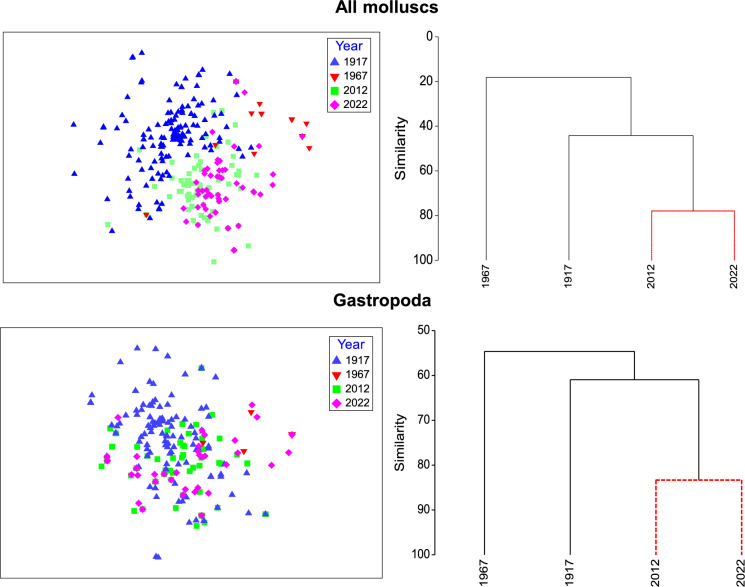
Non-parametric multi-dimensional scaling (NMDS) ordination plots of molluscan community excluding dreissenids and gastropod assemblages based on Bray–Curtis similarities of species presence-absence in samples collected in the Lower South Bay of Oneida Lake in 1917 (Baker 1918), 1967 (Harman and Forney 1970), 2012 (Karatayev et al. 2014), and 2022 (this study) (stress = 0.13). Right plots: cluster analysis (group average) built on Bray–Curtis similarity matrix of average yearly total mollusc (upper right) and gastropod (lower right) densities in 1917, 1967, 2012, and 2022. Black solid lines are significant at P = 0.002 (SIMPROF test)

During the first mollusc surveys in 1915–1917, phosphorous load into Oneida Lake was likely relatively low because Secchi depth exceeded 3.5 m and macrophytes were abundant down to 4 m (Fig. 3, Fitzgerald et al. 2016). Gastropods were represented by 29 species and higher taxa of which only one species (*Bithynia tentaculata* (Linnaeus 1758)) was exotic and two others (*Elimia semicarinata* (Say 1829) and *Viviparus georgianus* (Lea 1834)) were native transplants. Native species constituted 99% of the gastropod density lake-wide with *M. lustrica* being the most common species in the LSB, representing 36% of all individual gastropods sampled. No exotic bivalves were present in 1915–1917. Unionids were very abundant in the lake and were represented by 8 species. Similarly, sphaeriids were very diverse and common during Baker’s initial study, including large species of *Sphaerium* and *Musculium* that were never found during later surveys (Fig. 2). Thus, the LSB molluscan community in 1917 was significantly different from all other studies (P = 0.002, SIMPROF test, Figs. 3, 4, 5).

### Eutrophication depletes gastropods

Eutrophication increased in the lake in the mid-twentieth century (Fitzgerald et al. 2016) and was associated with a decline in the lake-wide diversity of native gastropods (excluding exotic but including native transplant species) by 14% by 1967–1968 and by 46% in 1992–1995 (Fig. 3, Online Resource 2). Several native species were lost, including the regionally rare *Acella haldemani* (Binney 1867), *Lymnaea stagnalis* (Linnaeus 1758), *Gillia altilis* (Lea 1841), and *Birgella subglobosus* (Say 1825). From these four species, only *B. subglobosus* returned as of 2022–2023. Two new-to-the-lake native transplant gastropods, *Elimia virginica* (Say 1817) and *Pleurocera canaliculata* (Say 1821), were recorded in 1967–1968.

The density of native gastropods in the LSB in 1967 declined on average by a factor of 23 (from 830 m^−2^ in 1917 to 37 m^−2^ in 1967), and this decline was evident across all families (Fig. 3). The density of previously dominant *M. lustrica* declined > 300-fold. Previously abundant Lymnaeidae and Physidae were not recorded at all in the LSB in 1967, while the density of exotic *B. tentaculata* in the bay increased 49-fold compared to 1915–1917. *Bithynia tentaculata* became the dominant species, representing 95% of all gastropods in the LSB. These changes were accompanied by strong alteration in the molluscan community (Figs. 4, 5).

Four unionid species recorded in the lake in the 1915–1917 (*Alasmidonta undulata* (Say 1817), *Pyganodon cataracta* (Say 1817)*, Cambarunio iris* (Lea 1829) and *Ortmanniana ligamentina* (Lamarck 1819)) were not found in 1967. At the same time, *M. margaritifera*, native to the region, was recorded for the first time along with three new native transplants: *Potamilus alatus* (Say 1817)*, Potamilus fragilis* (Rafinesque 1820), and *Ligumia recta* (Lamarck 1819). In contrast to gastropods, no change in the frequency of occurrence or lake-wide diversity (number of species) of unionids bivalves was detected in 1967, but the frequency of occurrence of *Pisidium* spp. in samples collected at the same sites declined from 62% in 1917 to 10% in 1967 (Karatayev et al. 2014). The combined diversity of native and introduced unionids in the lake did not change with eutrophication, though the diversity of native molluscs declined by 32%. The molluscan community in 1967–68 became significantly different compared to 1915–17 (Figs. 4, 5).

### Dreissenids extirpate unionids and increase gastropods

The introduction of the zebra mussel into Oneida Lake in 1991 had a strong impact on the whole ecosystem, including direct and indirect impacts on molluscs. The overgrowth of unionids by zebra mussels caused their extirpation in mid-1990 and they have not recovered as of 2023. While the frequency of lake-wide occurrence of *Pisidium* spp. had doubled in 2012 compared to 1967 (10%) and reached 21%, it was still threefold lower than in 1917 (62%).

Increased water clarity in the lake due to dreissenid feeding and filtering activities allowed periphyton and benthic algae to spread deeper into the lake, improving food resources for gastropods (Zhu et al. 2006; Cecala et al. 2008; Mayer et al. 2016). By 2012, diversity of all snails lake-wide increased by 88% compared to 1992–95 and became similar to 1915–17 (Table 1, Online Resource 2). However, there were changes in taxonomic composition: 5 species of native gastropods reported in 1915–17 were not found in 2012, whereas two new species not found in 1915–17 but native to New York State were first recorded in 2012. Finally, two exotic species (*Valvata piscinalis* (Muller 1774) and *Cipangopaludina chinensis* (Gray 1834)) were present in 2012, but not in previous lake-wide surveys.

**Table 1:** Long-term changes in species richness (exotic species richness in parenthesis) density (m^−2^) and wet biomass (g m^−2^) of molluscs in 1915–2023 in the Lower South Bay (LSB) and lake-wide (LW) in Oneida Lake. n.a.—data not available

Taxa	1915–1917 (Baker 1916, 1918)		1967–1968 (Harman and Forney 1970)		1992–1995 (Harman 1993, 2000)		2012 (Karatayev et al. 2014)		2022–2023 (This study)	
	LSB (1917)	LW	LSB (1967)	LW	LSB (1992–1995)	LW	LSB (2012)	LW	LSB (2022)	LW
*Gastropoda*										
Number of species	24 (1)	29 (1)	10 (1)	25 (1)	13 (1)	16 (1)	20 (2)	30 (3)	15 (2)	25 (2)
Density all	839 ± 128	n.a	472	n.a	n.a	n.a	746 ± 141	597 ± 123	226 ± 37	227 ± 55
Density exotic	8.9 ± 4.5	n.a	435	n.a	n.a	n.a	9.5 ± 4.6	60 ± 15	32 ± 11	34 ± 8
Biomass all	n.a	n.a	n.a	n.a	n.a	n.a	8.6 ± 1.4	6.2 ± 1.0	2.4 ± 0.5	2.9 ± 0.6
Biomass exotic	n.a	n.a	n.a	n.a	n.a	n.a	0.5 ± 0.2	2.1 ± 0.8	1.3 ± 0.3	1.9 ± 0.6
*Bivalvia*										
Number of species	7 (0)	11 (0)	n.a	8 (0)*	4 (1)*	6 (1)*	3 (2)	3 (2)	3 (2)	3 (2)
Density all	306 ± 104	n.a	n.a	n.a	n.a	n.a	2403 ± 610	2503 ± 457	2328 ± 452	2752 ± 558
Density of *D. polymorpha*	0	0	0	0	n.a	n.a	924 ± 194	699 ± 178	1254 ± 230	1047 ± 327
Density of *D. r. bugensis*	0	0	0	0	n.a	n.a	1463 ± 501	1765 ± 429	1057 ± 361	1619 ± 500
Density of dreissenids	0	0	0	0	n.a	n.a	2387 ± 610	2464 ± 461	2311 ± 452	2666 ± 563
Biomass all	n.a	n.a	n.a	n.a	n.a	n.a	678 ± 274	439 ± 118	112 ± 27	398 ± 139
Biomass of *D. polymorpha*	n.a	n.a	n.a	n.a	n.a	n.a	114 ± 36	51 ± 14	49 ± 13	94 ± 23
Biomass of *D. r. bugensis*	n.a	n.a	n.a	n.a	n.a	n.a	564 ± 264	388 ± 116	63 ± 22	303 ± 136
Biomass of dreissenids	n.a	n.a	n.a	n.a	n.a	n.a	678 ± 274	439 ± 118	112 ± 27	398 ± 139
Total species	31 (1)	40 (1)	10 (1)	33 (1)	17 (1)	22 (2)	23 (4)	33 (5)	18 (4)	28 (4)

^*^Excluding Sphaeriidae

Compared to the 1967 survey, density of all gastropods in the LSB in 2012 increased by 1.6-fold (Table 1). The largest increase was recorded for Amniculidae, Planorbidae and Physidae. Analyses of dominance curves, group clustering and NMDS revealed that the LSB gastropod community in 2012 was different from 1967 (P = 0.001) and became similar to 1917 (P > 0.90, SIMPER). *Gyraulus parvus* (Say 1817) became the most abundant species, representing 23% of all gastropod density in the bay excluding dreissenids. Density of exotic *B. tentaculata* declined > 46-fold. Density of Sphaeriidae (not recorded in 1967) declined in the LSB in 2012 compared to 1917 by a factor of 19. Abundant in 1917, *Sphaerium* spp. and *Musculium* spp. disappeared from the lake. Exotic dreissenids in 2012 exceeded the density of all other molluscs combined in the LSB by 3.1-fold and lake-wide by 3.9-fold. Regardless of these differences, the overall mollusc community (excluding dreissenids) in 2012 more closely resembled the pristine community in 1915–17 in terms of both diversity and density than the mollusc community in 1967–68 (P = 0.001, SIMPER, Figs. 4, 5). Thus, dreissenid mussels improved the conditions for gastropods (but not native bivalves) in the lake (Mayer et al. 2002; Karatayev et al. 2014).

### Round gobies reshape molluscan community

#### Gastropods swift decline and partial recovery (15 years of monitoring)

**Fig. 6:**
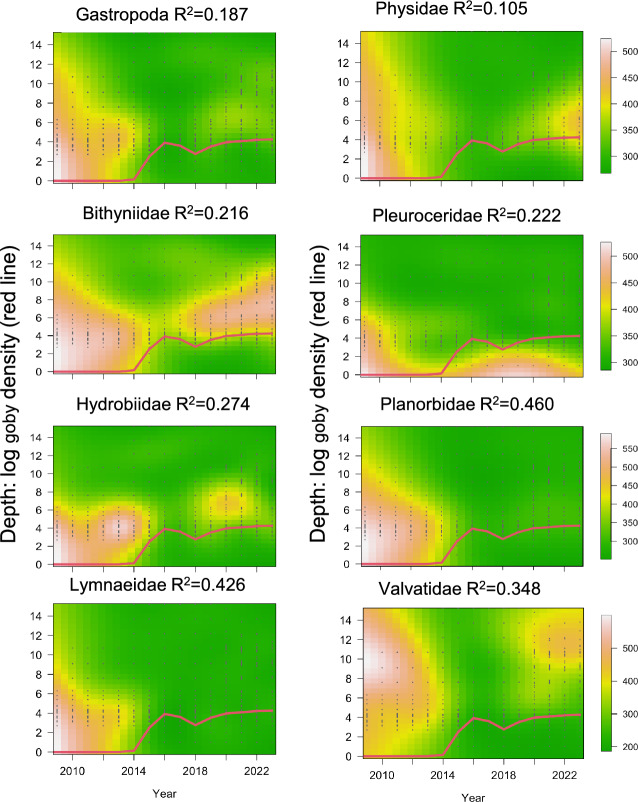
Dynamics of gastropod density in Oneida Lake across time and space. Colors are predictions of generalized additive models incorporating all effects by gastropod family (depth, year, and depth-year interaction) fitted to density. Red lines denote the relative change in lake-wide goby abundance index, log + 1 transformed. The number of sites included increase from 21 in 2009 to 60 in 2023. Before 2020, deeper sites were classified in depth regions and exact sample depth not retained. However, more than one sample within each depth dots is in water deeper than 8 m

Fifteen years of annual monitoring data revealed the timing and the magnitude of the round goby effect on gastropods (Fig. 6). A significant decline in gastropod density was recorded for all families. For every family, accounting for a linear effect of log (1 + goby density) (expressed as goby index) significantly improved model fit compared to models without goby density (i.e. AIC difference > 2, Online Resource 3). Considering that, in preliminary analyses, non-logged goby density achieved a much worse model fit, this indicates that molluscs declined at very low goby densities, potentially due to molluscs being a preferred food source for gobies.

**Fig. 7:**
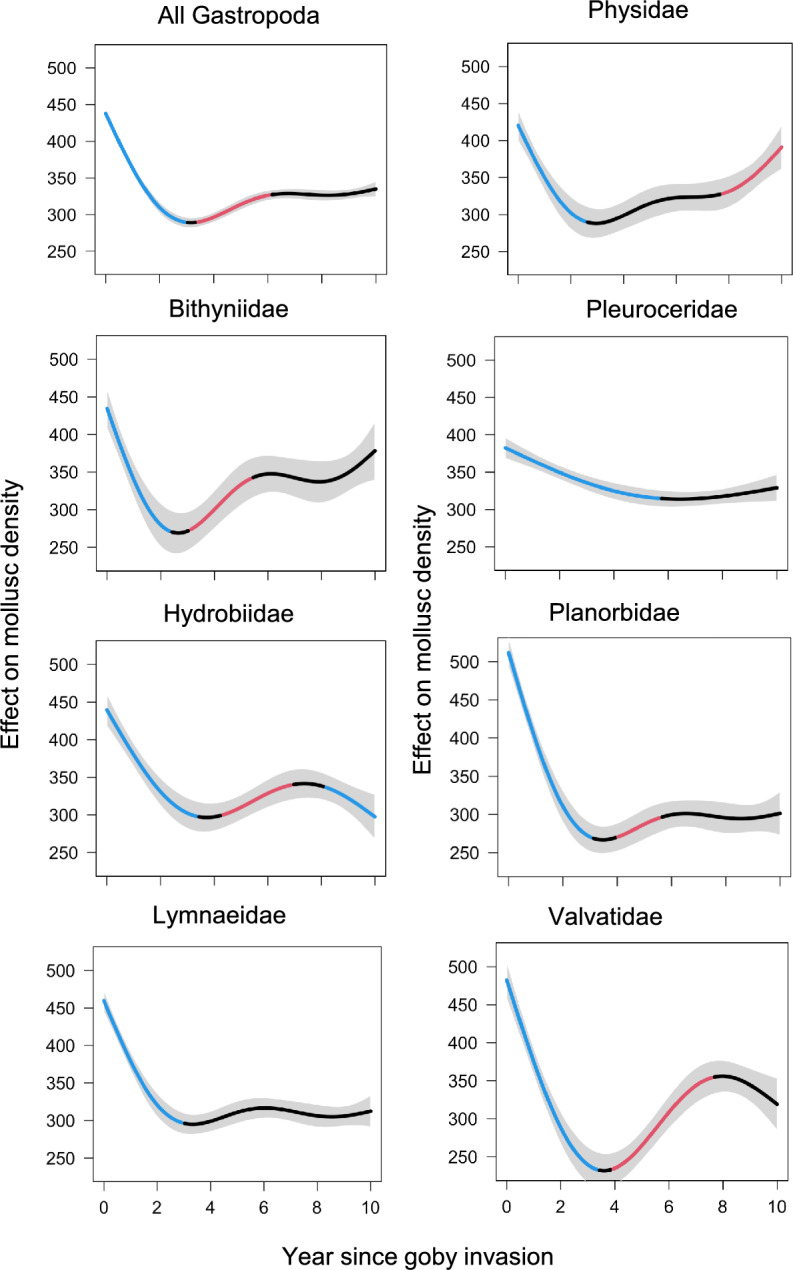
Round goby invasion corresponds with significant changes in gastropod density. Lines denote mean z-score changes in variables predicted by best-fit splines across years since invasion (x-axes) across all years without goby (that is, years without, or preceding, goby invasion). For each gastropod family, line colors denote significant increases (in red), significant decreases (in blue), no significant change (in black). Grey areas denote 95% confidence intervals of the mean. Note that only stations within > 2–11 m depth interval that were sampled through 2009–2023 are included

We also found evidence for a partial molluscan recovery from the goby invasion. Models with a nonlinear effect of year since goby invasion far outperformed models with a linear effect of goby density for 5 of 7 families considered, as well as for all gastropods combined. These nonlinear effects included a significant increase in mollusc density between years 3 and 7 since goby invasion (Fig. 7). The mollusc recovery, however, was limited to deeper depths. Reflecting these trends, models with an interaction between year and depth outperformed models without a year-depth interaction for all family groups.

#### *Round gobies deplete molluscs lake-wide* (2012 versus 2022–23)

The arrival of round goby in 2014 was associated with the 17% decline in the lake-wide gastropod diversity in 2022–2023 compared to 2012 (Fig. 3; Table 1). Four native species (*Stagnicola elodes* (Say 1821)*, Planorbella campanulata* (Say 1821)*, Gyraulus crista* (Linnaeus 1758), and *Laevepax fuscus* (Adams 1841)), one exotic (*C. chinensis*), and one native transplant species (*V. georgianus*) present in 2012 were not found in 2022–23, while *Probythinella emarginata* (Kuster 1852) was found in 2023 for the first time since 1915–17. However, all five species missing in 2022–23 were extremely rare in 2012. Therefore, it may be premature to make conclusions about changes in the total amount of species between the last two surveys, as these species could still be present in the lake but overlooked in surveys due to the low densities.

The average lake-wide density and biomass of all molluscs (including dreissenids) weighted by depth zones in 2012 were not significantly different from 2022–23 (density: 3100 ± 635 m^−2^ versus 2979 ± 377 m^−2^, P = 0.12; biomass: 445.2 g m^−2^ versus 400.9 g m^−2^, P = 0.22). However, these results were due to the lack of change in the density of the dominant dreissenid mussels. We did find a significant 2.6-fold decline in gastropod density (P < 0.001, t-test) and 2.1-fold decline in gastropod biomass (P < 0.001) (Table 1).

Goby predation had a strong impact on most native and nonnative species, but the impact was not uniform across major taxa and species (Figs. 3, 6, Online Resource 2). Density of all soft-shelled gastropods combined (Physidae, Lymnaeidae, Amnicolidae, Hydrobiidae, Valvatidae and small species of Planorbidae) in 2022–23 declined twofold and biomass fourfold (P < 0.001). The largest change was in the density of snails with shells of medium hardness (*Helisoma anceps* (Menke 1830)*, Planorbella trivolvis* (Say 1817)*, P. campanulata* and *B. tentaculata*), which declined 19-fold (P = 0.01). Predation was also size-dependent. Small soft-shelled snails (Amnicolidae, Hydrobiidae, Valvatidae and small species of Planorbidae) on average declined only 1.6-fold (P = 0.013) while medium size soft-shelled snails (Physidae, Lymnaeidae) declined sixfold (P < 0.001). Medium sized snails with medium shell hardness (*B. tentaculata*) declined 20-fold (P = 0.011). The largest decline was recorded for the medium size soft shell *S. catascopium,* whose density plummeted > 200-fold and biomass > 800-fold from 2012 to 2022–23. In contrast, the lake-wide density of large hard-shelled Pleurocerids in 2012 and 2022–23 was almost identical (15.1 ± 8.3 versus 17.6 ± 7.2 m^−2^, P = 0.82), but the biomass was almost twofold lower in 2022-23 (1.8 ± 1.0 vs 3.3 ± 1.0, P = 0.31). The average weight of all gastropods had changed two-fold from 0.032 ± 0.006 to 0.016 ± 0.003 g (P = 0.028). The average individual weight of some snails declined from 15-fold (*P. canaliculata*, P < 0.001) to twofold (*M. lustrica*, P = 0.004; *A. limosus*, P = 0.04; *Physella* sp. P = 0.001; and *G. parvus,* P = 0.008).

In contrast to gastropods, the impact of round gobies on dreissenids was lower. Lake-wide quagga mussel density (adjusted by depth areas) was similar in 2012 (1765 ± 429 m^−2^) and 2022–23 (1619 ± 500 m^−2^), although biomass somewhat declined (P = 0.07). Zebra mussel density increased 1.5-fold, and biomass 1.8-fold, but both changes were not significant (density P = 0.35, biomass P = 0.35; Table 1). The relative proportion of *D. polymorpha* increased both in terms of density (from 28% in 2012 to 39% in 2022–23) and in biomass (from 12% in 2012 to 24% in 2022–23).

### *Goby impact on mollusc depth distribution*

**Fig. 8:**
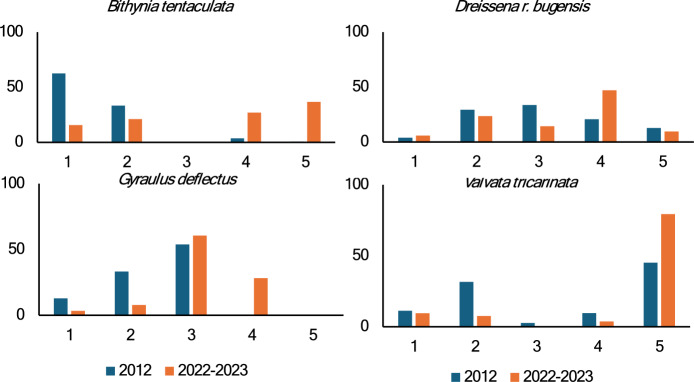
Changes in mollusc depth distribution after round goby invasion. The values represent the percentage of species density at each depth zone from the total. Depth categories on x-axis are 1: ≤ 0.5; 2: > 0.5–1; 3: > 1–3; 4: > 3–7; 5: > 7 m

We found a strong impact of round goby invasion on mollusc depth distribution between 2012 and 2022–23. The changes were significant for densities of molluscs that were fairly common in both years: *D. polymorpha* (P = 0.017, Fisher’s exact test), *D. r. bugensis* (P = 0.0006), *A. limosus* (P < 0.001), *B. tentaculata* (P < 0.001), *Gyraulus deflectus* (Say 1824) (P < 0.001), *G. parvus* (P = 0.008), *S. catascopium* (P < 0.001), and *Valvata tricarinata* (Say 1817) (P < 0.001) (Fig. 8). Changes were also significant for larger taxonomic groups: Lymnaeidae, Planorbidae, and Valvatidae (P < 0.001), and for all Gastropoda (P = 0.02). Most differences were due to the shift of molluscs deeper into the lake (Fig. 8).

## Discussion

Over the last hundred years the molluscan community of Oneida Lake was affected by numerous stressors, from eutrophication to multiple introductions of exotic molluscs and fish. Molluscan communities were sensitive to ecosystem change and invasive species, with some invaders offsetting the impacts of eutrophication and habitat alterations. The ability of zebra and quagga mussels to increase water clarity resulting in the increase in macrophytes, benthic algae, and periphyton has long been recognized (reviewed in Karatayev et al. 2002, 2015; Mayer et al. 2014; Higgins and Vander Zanden 2010), including in Oneida Lake (Mayer et al. 2016; Zhu et al. 2006; Karatayev et al. 2014). An increase in benthic primary producers resulted in the increase in gastropods previously diminished during the peak of eutrophication due to the declines in water clarity, benthic algae, and periphyton. In addition, dreissenid aggregations provide shelter and food resources for other benthic invertebrates (Mayer et al. 2002; Karatayev et al. 2007; Burlakova et al. 2012) (see below) and exotic and native mussels are used in several European and Asian waterbodies for biomanipulation purposes to decrease the effects of anthropogenic eutrophication (reviewed in Burlakova et al. 2023; Zhang et al. 2014). While individual stressors have taxon-specific and sometimes positive impacts, eutrophication and the serial invasion of first dreissenid mussels and then the round goby have collectively decimated the native mollusc community over the past century in Oneida Lake.

### Eutrophication depletes gastropods

As in many North American lakes, phosphorous loading in Oneida Lake increased in the mid-1900s due to watershed development, changing agricultural practices, and phosphorus in detergents (Fitzgerald et al. 2016). Combined with shoreline development and water level regulation (Mills et al. 1978), this eutrophication was associated with declines in macrophytes (Fitzgerald et al. 2006, 2016; Fig. 3). The highly diverse and abundant native gastropod community was dramatically reduced during this period (Karatayev et al. 2014; Fig. 3B), likely due to a decline in the primary food source—macrophytes, periphyton, and benthic algae (Mayer et al. 2016; Brown and Lydeard 2010) as a result of increased turbidity associated with eutrophication. *Bithynia tentaculata* became the dominant species in 1967, representing 95% of all gastropods sampled in the LSB. This species, unlike many native snails, can both graze and suspension feed, giving it a competitive advantage in eutrophic environments with abundant phytoplankton (Brendelberger and Jurgens 1993).

Sphaeriids experienced a dramatic decline along with the large-bodied *Sphaerium* and *Musculium* species which were extirpated and never returned. Eutrophication was most likely the reason for this decline, as many fingernail clams are sensitive to nutrient enrichment and associated low oxygen conditions (Holopainen and Jónasson 1983). Similar extirpations of previously abundant large sphaeriids in the 1970s was documented in Lake Mendota following eutrophication (Karatayev et al. 2013). Although the total number of unionid species remained unchanged (Table 1), native species diversity declined by 18% as four native unionid species disappeared. At the same time, one new native to the region species, *M. margaritifera*, along with three more native transplant species (*P. alatus, P. fragilis*, and *L. recta)*, were recorded in the lake in 1967–68 (Harman and Forney 1970). The three unionid species and two native transplant gastropods (*E. virginica* and *P. canaliculata)* were most likely introduced via the Lake Erie Canal System during 1920–1950 (Mills et al. 2016). As a result, by 1967–68, the molluscan community was very different compared to 1915–17 (Fig. 5). Altogether, the simplification of the community is evident in a much flatter dominance curve observed in the 1960s compared to steeper curves in all other years, indicating that the community during eutrophication was dominated by a few very abundant species (Fig. 4).

Following the improvement of sewage treatment facilities in the watershed, nutrient input to the lake began to subside in the late 1970s and phosphorus concentrations in the lake declined (Zhu et al. 2006; Fitzgerald et al. 2016). However, annual average Secchi depth increased only by about 10% from the 1960s to the late 1980s (Fig. 3), and by the early 1990s gastropod species richness lake-wide further declined by 36% from the 1960s and by 45% from 1915–1917 (Table 1). Overall, eutrophication had a strong negative impact on the diversity and density of gastropods and sphaeriids, while unionids were less affected.

Changes in water level regulation and increased shoreline development could also have contributed to the changes in the mollusc community. Improvement to the Caughdenoy Dam in 1951–52 allowed for active lake level manipulation, including a winter drawdown to limit ice damage to shoreline properties and to improve the ability of the lake to absorb the spring snowmelt and more stable water levels in the summer period. Water level manipulations may have affected wetlands and nearshore macrophytes that were decoupled from the lake due to lower spring water levels (Schneider et al. 2016), and winter drawdown will expose nearshore macrophytes to freezing conditions they may not tolerate (Fitzgerald et al. 2016). In addition, housing development around the lake continued, which often included shoreline hardening. Although these changes likely caused the observed decline in emergent macrophytes from the early 1900s to the 1970s, submerged macrophytes were likely less affected and may have even increased (Fitzgerald et al. 2016). Because our samples were restricted to within lake sites, we believe that these habitat changes are less important explanations for the decline in submerged macrophytes (and therefore molluscs) than the decline in light transparency associated with cultural eutrophication. The return of submerged macrophytes after the mussel invasion, with no changes in water level management and shoreline structures associated with increased water clarity, support this argument (Zhu et al. 2006; Karatayev et al. 2023, see below).

### Dreissenids extirpate unionids and increased gastropods

Invasive species are considered one of the largest threats to biodiversity (Wilcove et al. 1998; Dudgeon et al. 2006; Dudgeon 2019). Byssate bivalves (zebra and quagga mussels in the Northern hemisphere and the golden mussel *Limnoperna fortunei* (Dunker 1857) in the Southern hemisphere) are rapidly spreading across freshwaters and decimating native unionids, the most endangered group of freshwater animals (reviewed in Burlakova et al. 2000; Karatayev et al. 2007; Lucy et al. 2014; Karatayev and Burlakova 2022a). The introduction of *D. polymorpha* into Oneida Lake in 1991 was associated with a complete extirpation of unionids and substantial recovery of gastropods (Karatayev et al. 2014). The impact of *D. polymorpha* on unionids was swift and severe: most clams were already dead by 1993, only two years after the initial discovery of zebra mussels in the lake, and no living unionids have been found in surveys since 1995, including the 2012 lake-wide survey discussed above (Harman 2000; Karatayev et al. 2014) and the two benthic long-term data sets for Oneida Lake (Brooking et al. 2022). The negative impact of exotic *D. polymorpha* on native unionids is well documented in both Europe and North America (Burlakova et al. 2000; Lucy et al. 2014; Karatayev and Burlakova 2022a). However, Oneida Lake represents one of the most severe impacts of zebra mussel invasion, as in most of other waterbodies studied, *D. polymorpha* invasion usually did not lead to a complete extirpation of unionids and/or native clams recovered with time (Karatayev et al. 1997; Burlakova et al. 2000; Lucy et al. 2014). Interestingly, unionids are abundant in the tributaries of Oneida Lake (e.g. Chittenango and Fish creeks, authors pers. obs.). Dreissenids tend to have less dense populations in streams than in lakes as their larvae are swept downstream. The exceptions are rivers flowing from lakes or reservoirs populated by mussels (reviewed in Karatayev et al. 1998). Both Chittenango and Fish Creek are relatively small streams with virtually no zebra or quagga mussels present, thus serving as a refuge for unionids. Over several years we collected more than two hundred live unionids from these streams and observed only a single zebra mussel attached to a shell.

The *Dreissena polymorpha* invasion into Oneida Lake was associated with a significant increase in Secchi depth, macrophyte coverage, and the expansion of the maximum depth of submerged aquatic vegetation from 2–3 m in 1970s to 5–7 m in 2000s (Fig. 3; Zhu et al. 2006; Fitzgerald et al. 2016; Mayer et al. 2016). Similar changes in the water clarity and macrophyte coverage were widely reported in other waterbodies following *D. polymorpha* invasions (Karatayev et al. 1997, 2002; Higgins and Vander Zanden 2010; Mayer et al. 2014; Karatayev and Burlakova 2022a). By 2004, the primary production of benthic algae had increased significantly (Cecala et al. 2008; Mayer et al. 2016) resulting in the increase in shallow-water benthic grazers (amphipods and gastropods) by 1995, within 5 years of the first detection of *D. polymorpha* (Mayer et al. 2002, 2016).

By 2012 the diversity of gastropods in Oneida Lake had increased 88% compared to 1992–95 and became similar to 1915–17 (Table 1). Two new exotic species (*V. piscinalis* and *C. chinensis*) were present in 2012. *Valvata piscinalis* was recorded in Oneida Lake at 13 of 15 sites sampled in 2002 (Mills et al. 2016) and, according to Grigorovich et al. (2005), may have invaded the lake some time ago but was not recognized due to its resemblance to the native *Valvata sincera* (Say 1824). *Cipangopaludina chinensis* was noted in 1977 (Clarke 1978) but was not reported again until Karatayev et al. (2014) rediscovered it in 2012 (reviewed in Mills et al. 2016). Gastropod density in the LSB in 2012 increased 1.6-fold compared to 1967 and was similar to 1917 (Table 1). The largest increase was recorded for Amniculidae, Planorbidae and Physidae. As a result, cluster and NMDS analyses revealed that the 2012 LSB gastropod community was different from 1967 (P = 0.001, SIMPER), but became more similar to 1917 (Fig. 5).

The recovery of gastropod abundance and species richness was primarily driven by an ecosystem-wide increase in water clarity resulting in a substantial increase in macrophyte coverage and benthic and periphyton algae production, which expanded the main energy source of benthic grazers. In addition, several local-scale dreissenid impacts on benthic grazers could have contributed to this recovery. Dreissenids can facilitate benthic grazers, including gastropods, by changing bottom substrates and creating new three-dimensional reef-like structures that provide refuge from predation and other stressors, by food resources provided by the mussels (e.g. feces and pseudofeces), and associated organisms including abundant periphyton on mussel shells (Botts et al. 1996; Stewart et al. 1998; Karatayev et al. 1997, 2002, 2007; Mayer et al. 2002; Higgins and Vander Zanden 2010; Burlakova et al. 2012). Overall, the introduction of dreissenids had strong taxa-specific effects on Oneida Lake molluscs, including extirpation of unionids and recovery of gastropods. Dreissenids became dominant, forming 80% of the density and 99% of the biomass of all molluscs in Oneida Lake (Table 1).

### Gobies deplete gastropods

The arrival of goby in 2014 had a strong negative impact on virtually all gastropod species in Oneida Lake as well as quagga mussels. The impacts manifested quickly within the first 2–3 years since the initial invasion (Figs. 6, 7). This decline was documented in Brooking et al. (2022). As a benthic feeder, the round goby actively feeds on bottom invertebrates, including various molluscs and dreissenids (Johnson et al. 2005; Lederer et al. 2006, 2008; Poslednik et al. 2023; Karatayev et al. 2024). Therefore, the observed decline in molluscan density in Oneida Lake was not surprising. It was surprising, however, to find a partial significant recovery in most gastropods between years 3 and 7 since goby invasion (Fig. 7). Another intriguing discovery was that mollusc recovery was depth-related, being limited to deeper areas (Fig. 6). Although samples from depths  < 2 m were limited in the dataset we used to generate depth distribution model, other studies conducted in 2010 (unpublished data) and 2012 (Karatayev et al. 2014) found the highest gastropod diversity and abundance at  < 2 m. This supports model projections at these depths. We note that the round goby is most abundant in shallower water and rocky substrate, although they are found throughout the lake (VanDeValk et al. 2024). This dynamic of goby impacts on gastropods in Oneida Lake, from a sharp decline in density followed by a significant recovery in areas with lower goby abundance, was possible to reveal only due to annual sampling of benthos conducted during 2009–23.

Comparison of the large-scale surveys conducted before (2012) and 8–9 years after the initial goby invasion (2022–23) revealed that goby impact on gastropods was not uniform across major taxa and species (Figs. 3, 7). As we predicted, the strongest decline was recorded for soft-shelled species with Lymnaeidae and *Physella* spp. being affected the most, regardless of the partial recovery of the latter group (Fig. 6). In contrast, the density of large hard-shelled pleurocerids, although initially declined (Fig. 7), had completely recovered by 2022–23 and was not significantly different from 2012 (15.1 versus 17.6 m^−2^, P = 0.82). The goby impact on gastropods was also size-dependent, with medium and large soft-shelled snails being more affected than small soft-shelled snails. This is not surprising as it was shown that *Dreissena* sizes preferable to gobies consumption is about 4–13 mm (Naddafi and Rudstam 2014a, b; Poslednik et al. 2023), and almost all our most common small gastropods were < 4 mm (Jokinen 1992).

Changes in snail behavior and depth distribution in response to fish and invertebrate predations are well documented with the medium and soft shelled (e.g. *Physella*) gastropods being affected the most, whereas large thick shelled species (e.g. *Planorbella*) are least affected (Alexander and Covich 1991a, b; Turner et al. 2000; Turner and Montgomery 2003). Snails change behavior and use cover and other hiding places in the presence of predators (Alexander and Covich 1991a, b; Turner 1996; Turner et al. 1999; Turner and Montgomery 2003) resulting in less efficient grazing of periphyton (McCollum et al. 1998; Turner et al. 2000), slower growth rates and smaller sizes (Turner and Montgomery 2003), and smaller sizes at reproduction (Crowl and Covich 1990). The strong impact of goby invasion on distribution, community structure, and mollusc sizes we observed in this study was most likely due to the slower mollusc growth rate as a result of the cost of predator avoidance behavior. This is supported by the lower average weight of virtually all gastropods (P = 0.03) in 2022–23 compared to 2012, indicating that goby predation may have a size-selective effect both on the community level (disproportionately higher decline of medium and large-bodied snails) and on population level (decline in the average weight of snails).

The round goby invasion resulted in the increase in zebra mussel relative abundance lake-wide, but the difference was depth-dependent and declined with depth. Round gobies are adapted to feed on dreissenids through coevolutionary history (Kornis et al. 2012; Naddafi and Rudstam 2014a; Rudstam and Gandino 2020) and are known to impact the density of mussels both locally and lake-wide (Barton et al. 2005; Patterson et al. 2005; Rudstam and Gandino 2020). Fish in general and round goby in particular prefer quagga mussels over zebra mussels when they are of the same size (Naddafi and Rudstam 2014b) and the increase in gobies should therefore increase the relative density of zebra mussels. This was observed in a nearby lake after goby invasion (Rudstam and Gandino 2020) and also documented in the Ponto-Caspian region (Zhulidov et al. 2006, 2010). Quagga mussels are more vulnerable to predation because their shells are thinner, their attachment strength is lower, their aggregation behavior is weaker, and they are less prone to seek refuge (reviewed in Karatayev and Burlakova 2022a).

Overall, round gobies had a strong impact on the molluscan community of Oneida Lake. The impact depended on mollusc species, shell strength and depth. The maximum impact occurred within the first 2–3 years after invasion and was followed by partial recovery in deeper waters. The strong impact early in the invasion when the biomass of a new introduced species is growing rapidly, followed by partial recovery, is not uncommon (reviewed in Strayer et al. 2006; Karatayev et al. 2023). Population abundance and impacts of an invader often exhibit an invasion cycle where, after initial high abundance, invader populations decline (Simberloff and Gibbons 2004; Karatayev et al. 2015; Strayer et al. 2017) due to changes in community structure, density-dependent changes in the abundance of the invader, evolutionary and behavioral adaptations by resident species to the new invader, or other factors (Karatayev et al. 2023). As goby density in Oneida Lake over the study period did not decline (Fig. 5), the partial recovery of the molluscan community was most likely due to changes in the species composition, including a sharp decline in medium and large size soft-shelled species as well as behavioral adaptation of molluscs by moving into deeper areas where goby density is lower (Brooking et al. 2022).

### Multiple invasions amplify negative impact

Introductions of keystone or ecosystem engineering species can profoundly transform multiple ecosystem features (reviewed in Hui and Richardson 2017). The response of ecosystems to a species introduction can be complex and difficult to predict, especially if multiple species are introduced in series (for example, invasion meltdown, Simberloff and Von Holle 1999). Potentially, the ecosystem impacts of serial invasions range from minimal ecosystem change to an amplification of the initial invader’s impacts and a prevention of ecosystem recovery (reviewed in Karatayev et al. 2023). Shifting baselines when every new stressor is impacting ecosystems already affected by previous stressors make serial invasions especially challenging for monitoring and risk assessment, and lead to difficulties in selecting a baseline for restoration (Guerrero-Gatica et al. 2019). Each of the stressors considered in this paper affects a large number of waterbodies worldwide. Zebra mussels in the USA alone already colonized 1230 waterbodies as of 2021 (Karatayev and Burlakova 2022a), and the round goby is rapidly spreading across Europe and North America. By feeding on invasive dreissenids and native molluscs (Kornis et al. 2012), the round goby strongly affects benthic invertebrates already imperiled by dreissenids and other stressors in multiple lakes (reviewed in Karatayev et al. 2022). Round gobies may have a larger effect on native molluscs than native benthivores due to the established dreissenid populations. The round goby is an efficient feeder on dreissenid mussels and can therefore maintain higher populations in lakes with dreissenids, like Oneida Lake, than native benthivores.

We found that over the past hundred years, the molluscan community of Oneida Lake experienced the effects of eutrophication and multiple invasions, including zebra and quagga mussels, as well as round goby. Overall, the impacts of multiple stressors resulted in declines in species diversity and density of native gastropods and extirpation of unionids, *Sphaerium* spp. and *Musculium* spp. Dreissenid mussels currently comprise 89% of density and 99% of wet biomass of all molluscs in Oneida Lake. While individual stressors have taxon-specific and sometimes positive impacts, eutrophication and species invasions have collectively decimated the native mollusc community over the past century.

## Supplementary Information

Below is the link to the electronic supplementary material.

Supplementary file1 Online Resource 1: Density and biomass of molluscs in Oneida Lake in 2012-2023.

Supplementary file2 Online Resource 2: Excel file with Species composition of molluscs in Oneida Lake in 1915-2023. Original and accepted (according to Integrated Taxonomic Index System (http://www.itis.gov) species names for the current paper are given.

Supplementary file3 Online Resource 3: Comparison of model fit for models accounting for depth, year, and goby effects.

## Data Availability

Data are provided in Supplementary Information Online Resources 1–3.
