# Supplementary material for: Multiple invasions decimate the most imperiled freshwater invertebrates
Source: Biol Invasions. 2025 Feb 13;27(2):85. doi: 10.1007/s10530-025-03540-5 (PMC11821755; doi:10.1007/s10530-025-03540-5)
Supplement: Supplementary file 3 — Supplementary file3 Online Resource 3: Comparison of model fit for models accounting for depth, year, and goby effects. [file 10530_2025_3540_MOESM3_ESM.docx]

Online Resource 3.

Increases in R^2^ and decreases in AIC for models in our analysis compared to models with an intercept only. In model names, "D" = models with depth only, "Y" = models with year only, "G" = models with a linear effect of gobies, "YSG" = models with an effect of year since goby invasion, and "YD" = models with an interaction between depth and year. Except for Glin, all models were 1d or (for YD) 2d splines of predictors.

| Family | YD | Y+D | Y | YSG | Glin | D | none |
| --- | --- | --- | --- | --- | --- | --- | --- |
| 1 | 0.179 | 0.203 | 0.129 | 0.084 | 0.044 | 0.092 | 0 |
| 2 | 0.178 | 0.17 | 0.155 | 0.09 | 0.072 | 0.043 | 0 |
| 3 | 0.339 | 0.363 | 0.298 | 0.238 | 0.154 | 0.033 | 0.001 |
| 4 | 0.257 | 0.286 | 0.254 | 0.209 | 0.166 | 0.082 | 0 |
|  |  |  |  |  |  |  |  |
| 1 | 99 | 97 | 63 | 49 | 27 | 43 | 0 |
| 2 | 105 | 104 | 95 | 53 | 45 | 26 | 0 |
| 3 | 227 | 234 | 188 | 160 | 100 | 10 | 0 |
| 4 | 696 | 775 | 686 | 563 | 438 | 191 | 0 |

.

Families:

1 = Bithyniidae

2 = Pleuroceridae

3 = Valvatidae

4 = "Lymnaeidae" "Physidae" "Hydrobiidae" "Planorbidae" combined
